# Effects of feast-famine nutrient regimes on wastewater algal biofuel communities

**DOI:** 10.1371/journal.pone.0279943

**Published:** 2023-01-04

**Authors:** Mark H. Loria, James S. Griffin, George F. Wells, Kurt R. Rhoads

**Affiliations:** 1 Department of Civil and Environmental Engineering, Case Western Reserve University, Cleveland, Ohio, United States of America; 2 Department of Civil and Environmental Engineering, Northwestern University, Evanston, Illinois, United States of America; Doctor Harisingh Gour Vishwavidyalaya: Dr Hari Singh Gour University, INDIA

## Abstract

Microalgae accumulate lipids in response to nutrient deprivation, and these lipids are a biodiesel fuel stock. Algal cultivation with secondary wastewater effluent is one proposed platform for biofuel production, which provides nutrients to algae while further polishing wastewater effluent. Algal bioreactors were tested using a feast-famine feeding regiment in simulated secondary wastewater effluent to evaluate the effects on lipid content and algal community structure. Algal polycultures were inoculated into reactors fed with synthetic secondary wastewater effluent at pH 7.5 and 9 and operated under a feast-famine nutrient (N, P, and BOD) supply regime in sequencing batch reactors. Fatty acid methyl ester contents of the reactors were assessed, which showed a decrease in lipid content after the feast-famine cycling (from 12.2% initially to 5.2% after four cycles at pH 9). This decrease in lipid content was not correlated with an increase in carbohydrate storage within biomass, nor an increase in bacterial biomass abundance relative to algal biomass in the reactors. The eukaryotic microbial communities from reactors operated at pH 9 diverged from reactors operated at pH 7.5 during cycling, with the pH 9 reactors becoming dominated by a single Operational Taxonomic Unit aligning to the *Scenedesmus* genus. These results suggest that high pH and feast-famine nutrient cycling may select for a less diverse algal community with a lower lipid content within a secondary wastewater polishing scheme.

## Introduction

Algae can accumulate lipids which serve as a biodiesel feed stock, representing a potential source of alternative energy. Additionally, algae have a high growth rate and a higher productivity per land area used when compared to other biofuel feedstocks such as soybean [1]. Despite favorable aspects of algal biofuel production, remaining challenges include lowering cost, mitigating fertilizer and water requirements, and ensuring bioreactors remain stable and productive [2]. Thus, the economic viability of algal biofuels depends on the cost-effective cultivation of microalgal biomass without excessive nutrient or energy demands. One option for low-cost algal cultivation is to grow algae on secondary wastewater effluent [3–5]. With this approach, the wastewater itself provides nutrients (nitrogen (N), phosphorus (P), and biochemical oxygen demand (BOD) to microalgae, avoiding the need for direct fertilization. Additionally, such reactors could facilitate wastewater polishing, enhancing N and P removal from effluent through algal uptake prior to discharge to receiving water bodies. Given these benefits, this study assessed algal cultivation in reactors that replicate growth in secondary wastewater effluent.

In addition to examining algal growth on secondary treated wastewater effluent, this study also evaluated the effects of feast-famine nutrient cycling on the inoculated natural phytoplankton community. Feast-famine conditions occur when the microbial community is subject to cycling periods of high nutrient (N, P, and BOD) availability (i.e., feast) followed by low nutrient availability (i.e., famine). This approach has been demonstrated as an engineering paradigm for selecting for desirable traits within a microbial community, such as the use of feast-famine cycling for optimizing production of the storage polymer polyhydroxybutyrate (PHB) in bacteria [6]. Additionally, this cycling is the underlying mechanism in selecting for phosphate-accumulating organisms in enhanced biological phosphorus removal processes for wastewater treatment [7]. Thus, a possible benefit of feast-famine cycling on algal biomass could be an analogous selection of high lipid-accumulating microalgae, which would make the biomass more favorable for biodiesel production. Natural phytoplankton communities also experience intermittent pulses of nutrients (i.e., feast-famine) in vernal pools and tidal marshes [8, 9], so the effects of feast-famine cycling on algal-microbial communities also has implications for natural community ecosystem stability and function. While there are several possible reactor configurations for the large-scale production of algal biomass from secondary wastewater, including continuously-fed plug flow, completely mixed, or step feed reactors, sequencing batch reactors (SBRs) were selected for this study. Given the intermittent feeding associated with SBR operation, cultivation in SBRs can be used to readily impose a feast-famine nutrient supply regime on algal biomass, compared to continuously fed reactor options, which provide steadier nutrient conditions. Additionally, SBRs are well-suited to maintaining the high solids retention times (SRTs) anticipated for a photosynthetic microbial community (6 days or more) [10], and offer the potential to modify operation to encourage biomass granulation [11].

Conventional activated sludge wastewater treatment reactors are commonly maintained in a neutral pH range of 6.5 to 8.2 for effective treatment, depending on the specific process configuration [12]. Photosynthesis naturally increases the pH of surrounding media, however, and algal ponds treating wastewater have demonstrated high operating pH values, up to 9 or 10 under some cultivation conditions [13–15]. For this reason, this study evaluated the effects of pH on the cultivation of algal consortia by comparing reactors operated at a neutral pH 7.5 versus elevated pH 9. Operating algal biofuel reactors at an elevated pH could provide additional benefits for wastewater polishing, such as the attenuation of human pathogens in wastewater, which can occur at high pH values [16]. Additionally, successful operation at pH 9 or above can reduce the efforts and costs associated with buffering photosynthetically-induced pH changes. Given the natural inclination of algal bioreactors to operate at high pH, and the possible benefits of this for wastewater treatment, the effects of pH on lipid and biomass yields are also of interest.

The overall objective of this study was to investigate the algal community composition and lipid content of microalgae grown under a feast-famine feeding regime in SBRs operated at two different pH values under conditions reflecting feeding with secondary wastewater effluent. Reactors were intermittently fed with synthetic wastewater at pH 7.5 and pH 9 to examine the effects of a feast-famine operating scheme in a platform where algae are used for simultaneous wastewater polishing and biofuel production. Changes in the overall lipid and starch content of biomass, along with changes in fatty acid methyl ester (FAME) profiles, were monitored in conjunction with microbial community composition over reactor cycles to assess the effects of feast-famine cycling on lipid content of cultures and to determine the possible mechanisms underlying these effects.

## Materials and methods

### Media and culture inoculum

Microbial cultures were inoculated with water from Wade Lagoon in Cleveland, OH, in November 2014 obtained with permission of the lagoon owner. Cultures were grown using a modified WC Medium [17] with nutrient levels adjusted to approximate wastewater effluent after nitrification and COD removal (10 mg-N/L supplied as NO_3_^-^, and 1.1 mg-P/L supplied as PO_4_^3-^) [18] and with the following modifications: added 26.5 mg/L sodium acetate trihydrate to provide BOD, added 1 mg/L H_3_BO_3_, and excluded sodium carbonate and vanadium. Cultures were maintained at pH 7.5 or pH 9. For cultures maintained at pH 7.5, the media was buffered with 715 mg/L HEPES [19]. For cultures maintained at pH 9, the media was buffered with 730 mg/L TAPS.

### Reactor operation

Six 1-L sterile glass reactors were operated in a controlled environment plant growth chamber (Conviron A100, Canada) under 14-h daily illumination with 400 μmol m^-2^ s^-1^ photosynthetically active radiation (PAR), at 25°C. Three of these reactors were operated in replicate at pH 7.5, and the other three reactors were operated in replicate at pH 9. Reactors were mixed with a magnetic stir bar at approximately 120 rpm and were bubbled with filtered air at 0.22 L min^-1^ to facilitate gas exchange. Feast-famine nutrient cycling was imposed on reactors by repeating the following cycle: After 7 d of growth (for uptake of available N, P, and BOD), 450 mL of culture was removed, and cells were separated from media by centrifuging at 7400 × *g* for 10 min at 4°C. The resultant concentrated biomass was re-suspended in fresh media (with replete N, P, and BOD) to allow for another 7 days of growth. Tests showed that centrifuging monocultures of eukaryotic algae at this speed did not inhibit exponential cell growth. Cultures were initially inoculated with 50 mL of water sampled from Wade Lagoon into two 1-L reactors at pH 7.5 and 9 and pre-cultured to create an acclimated microalgal-microbial community. These starter cultures were used to inoculate the six experimental reactors (50 mL of pre-culture volume into the experimental reactors) and the experimental reactors were allowed to grow for 4 d before experimentation began. Reactor cycle 0 represents the first resuspension of biomass from the six experimental reactors into fresh media. Cycles 1, 2, 3, and 4 represent biomass which was sampled and then re-suspended after a 7-d growth period (batch operation). Total biomass in the reactors was tracked over time using total suspended solids (TSS) analysis (analogous to a mixed liquor suspended solids concentration) [20].

### Carbohydrates

Storage carbohydrates were analyzed using the Megazyme Total Starch Assay Kit (AA/AMG) (product number K-TSTA–Megazyme, Wicklow, Ireland). Total starch was extracted according to the manufacturer’s protocol for samples with resistant starch and D-glucose and/or maltodextrins, with modifications to reagent volume in proportion to the mass of algal sample extracted for analysis (approximately 5 mg). This kit was previously shown to be suitable for starch extraction from microalgae [21], but it was retested for repeatability with mixed microbial-algal material and at downsized reaction volumes (S1 Table and S1 Fig). Starch content was normalized to lyophilized weight of algae initially used for the starch extraction.

### Lipids

Lipid content of algae normalized to dry weight was quantified by analyzing fatty acid methyl ester (FAME) content for samples collected at the end of each 7-day growth cycle. FAMEs were extracted after an *in situ* transesterification of ~5 mg lyophilized algal biomass, according to a method developed by the National Renewable Energy Laboratory [22] (with minor modifications). The transesterification reaction was carried out in a water bath at 85°C after the addition of 300 μL of acidified methanol, 200 μL of 2:1 (v/v) chloroform: methanol, and 0.2 mg of tricosanoic acid methyl ester internal standard (PN 35044 –Restek, Bellefonte, PA) to lyophilized biomass. FAMEs were then partitioned into 1 mL of hexane.200 μL of this hexane phase was then mixed with 5 μg of pentadecane as an internal standard (PN 76509-5ML–Sigma-Aldrich, St. Louis, MO) and quantified by Gas Chromatography/Flame Ionization Detection with 37 FAME mix as a calibration standard (PN 18919-1AMP–Sigma-Aldrich, St. Louis, MO). GC/FID separation and detection was carried out using a J&W DB Wax column (PN 122–7032 –Chem-Agilent, Santa Clara, CA), with 1-μl injection volume (1:10 split ratio) and an inlet temperature of 253°C. The oven temperature profile was: 100°C for 1 min, 25°C/min to 200°C with 1 min hold, 5°C/min to 250°C with 7 min hold. The helium flow rate was 1ml/min, and FID conditions were: 280°C, 450 mL/min air, 40 mL/min hydrogen, and 30 mL/min helium. Calculated FAME concentrations were normalized to initial lyophilized algal biomass weight for total normalized lipid content determination. Samples were stored after lyophilization at -80°C, and analyzed for FAMEs in batches of six samples, chosen randomly from all samples from the study.

### Community sequencing analysis

Genomic DNA was extracted from replicate 1.5-ml aliquots of mixed biomass using the FastDNA Spin Kit for Soil (MP Bio, Santa Ana, CA). Following DNA extraction, genomic DNA concentration was measured via fluorescence using the Quant-It dsDNA quantification kit (Thermo-Fisher Scientific, Rockford, IL) on a 96-well plate reader (BioTek Instruments, Winoovski, VT). As a proxy for total bacterial abundance, 16S rRNA gene copies in extracts were quantified via qPCR and normalized to nanogram of genomic DNA. For each sample, two 20-uL qPCR reactions were performed using the Eub519F and Eub907R primer set [23] in a Biorad CFX Connect thermal cycler (Bio-Rad, Hercules, CA). Bio-Rad iQ SYBR Green Supermix (Bio-Rad, Hercules, CA, USA) was used for all qPCR assays, with DNA template ~1 ng and primer concentrations of 0.2 uM. Reaction conditions were 95°C for 5 minutes followed by 40 cycles of: 95°C (30 s), 55°C (45 s), and 68°C (30 s) and a final elongation step at 68°C for 7 min.

Gene (V8-V9) amplicon sequencing libraries of 18S rRNA were prepared using a two-step PCR amplification protocol. In the first PCR reaction, sequences were amplified using the V8F-1510R primer set [24] tagged with “common sequences” on each primer. Each 20-uL reaction contained 10 μL of FailSafe PCR 2X PreMix F (Epicentre, Madison, WI), 0.63 units of Expand High Fidelity PCR Taq Enzyme (Sigma-Aldrich, St. Louis, MO), 0.3 μM of forward primer and reverse primer modified with Fluidigm common sequences at the 5’ end of each primer, ~1 μL of gDNA (approximately 100 ng) and the remaining volume molecular biology grade water. The PCR conditions were 95°C for 3 min, followed by 25 cycles of 98°C for 20 s, 65°C for 15 s, and 72°C for 15 s, with a final extension at 72°C for 10 min. For the second round of PCR, round 1 PCR product from each sample was amplified using a unique barcoded primer (Fluidigm, San Francisco, CA). Conditions were 95°C for 5 min, followed by 8 cycles of 95°C for 30 s, 60°C for 30 s, and 68°C for 30 s, with a final extension at 68°C for 7 min. A negative PCR control with no template DNA was included for each round of PCR. DNA sequencing was performed using a MiSeq System (Illumina, San Diego CA) at the University of Illinois Chicago DNA Services Facility.

The 250 base pair, paired-end 18S rRNA gene amplicon reads were merged using PEAR [25], then quality filtered to remove reads with >1 error per 100 nucleotides, checked for chimeras and clustered into Operational Taxonomic Units (OTUs) using Vsearch [26]. Sequences were clustered into 199 non-chimeric OTUs that were used for downstream analysis. Taxonomy assignment was performed in QIIME [27] using the Silva Eukarya database (v108). To assess differences between samples in terms of composition, pairwise Weighted Unifrac distances were calculated from rarefied OTU abundance counts using QIIME’s “beta_diversity.py” script. Shannon Index was calculated using the Vegan R package [28]. OTUs were clustered at 97% similarity and putative taxonomy of OTUs was determined by NCBI BLAST alignment [29].

## Results and discussion

### Lipid content of microbial communities

**Fig 1:**
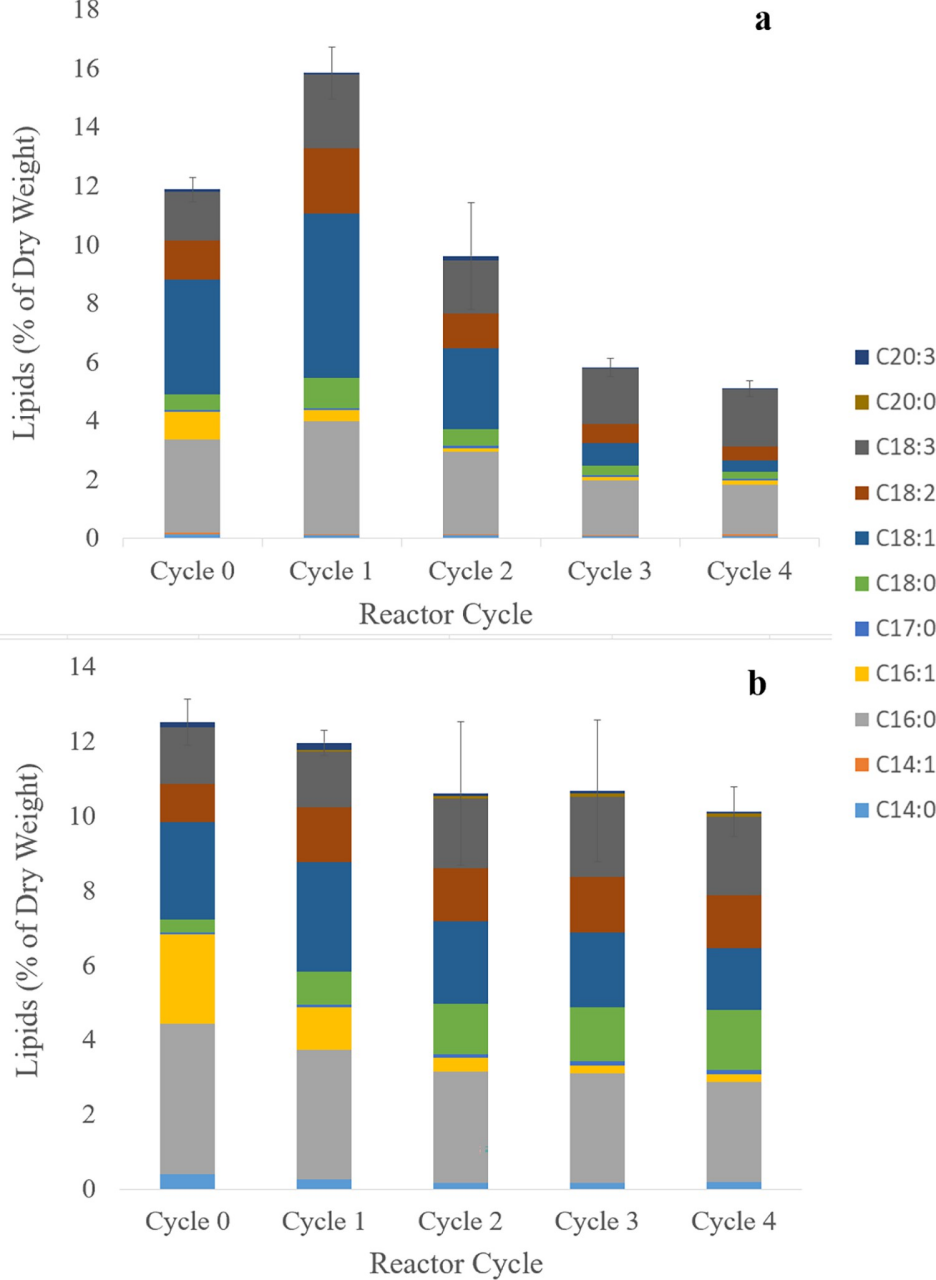
Lipid storage in algal biomass. Overall lipid content and FAME composition of a) pH 9 and b) pH 7.5 reactors over the course of feast-famine cycles. Error bars represent one standard deviation above and below of three biological triplicates.

After the first feast-famine reactor cycle, the lipid content of pH 9 reactors increased from 12.2 ± 0.42% to 16.2 ± 0.89%. The lipid content then decreased during subsequent cycles to a final average lipid content of 5.2 ± 0.27% (Fig 1). Compared to the pH 9 reactors, the lipid content of the pH 7.5 reactors varied less between feast-famine cycles (<1.5% week-to-week fluctuations), with a net decrease in average lipid content from 12.9 ± 0.62% to 10.3 ± 0.67% over four cycles (Fig 1). In the pH 9 reactors, the mean lipid content for each reactor cycle was significantly different than that of the subsequent cycle, except between cycles 3 and 4, according to ANOVA and Tukey Pairwise Comparisons (95% confidence). In contrast to the pH 9 reactors, ANOVA showed that the overall lipid content for the pH 7.5 reactors did not change significantly between cycles (P = 0.136). The pH 7.5 reactors had a significantly higher average lipid content (10.3%) at the end of operation compared to the pH 9 reactors (5.2%) (two sample t-test, equal variance, two-tail P = 0.0002).

These results indicate that feast-famine operation had a neutral to depressive effect on lipid-accumulation in microalgae within the reactors, and that pH 9 reactors responded to feast-famine cycling differently than pH 7.5 reactors. One possible explanation for the steady or decreasing biomass lipid content is that lipid content of the initial inoculum was low (average 12.2% and 12.9% for pH 9 and pH 7.5 reactors, respectively), indicating a limited number of lipid-accumulating species at the start of reactor operation. A previous study of mixed microalgal-microbial consortia reactors showed initial biomass lipid contents of 19–21% of dry weight after a pre-culture period on primary treated wastewater [13]. Cultures in this study started with a lower lipid content, and then feast-famine cycling further reduced this lipid content to 5.2% and 10.3% of dry weight at the end of reactor operation.

While the lipid content in the pH 7.5 reactors remained relatively constant, the lipid content in the pH 9 reactors decreased. Though the initial lipid composition profiles for both sets of reactors (pH 7.5 and pH 9) were similar, these profiles diverged from one another as a result of feast-famine cycling. Both sets of reactors initially showed a fatty acid profile consisting primarily of C16:0, C16:1, C18:1, C18:2 and C18:3 (Fig 1). However, the fatty acid profiles of each set diverged during feast-famine cycling, indicating a change in the microbial communities. The average C16:0 and C16:1 content decreased in the pH 7.5 reactors as a result of feast-famine cycling (by 1.3% and 2.2% of dry weight, respectively), along with an increase in average C18:0 content (by 1.3% dry weight). Reactors operated at pH 9 showed the largest decreases in average C18:1 and C16:0 content (by 3.5% and 1.5% dry weight, respectively), which accounted for 72% of overall lipid content change (Fig 1). The fatty acid profiles in pH 9 and pH 7.5 reactors initially had similar lipid profiles. After cycling, however, pH 7.5 reactors had a relatively even composition of predominantly C18:3, C18:2, C18:1, C18:0 and C16:0 (together accounting for 9.6% of the total 10.2% dry weight of lipids in biomass), whereas pH 9 reactors were composed of primarily C18:3 and C16:0 (together accounting for 3.6% of the total 5.1% dry weight of lipids in biomass).

### Starch content of microbial communities

Algae use starch as well as lipids as energy storage polymers. Therefore, starch was measured in the biomass to examine the possibility that feast-famine cycling increases starch content rather than lipid content in the cultivated biomass. The length of each feast-famine cycle was chosen to be 7 d, which coincides with studies indicating a maximum lipid accumulation peak in algae around 7 d after nutrient limitation for mixed-microalgal communities, whereas maximum starch accumulation occurred around 48 h [30].

**Fig 2:**
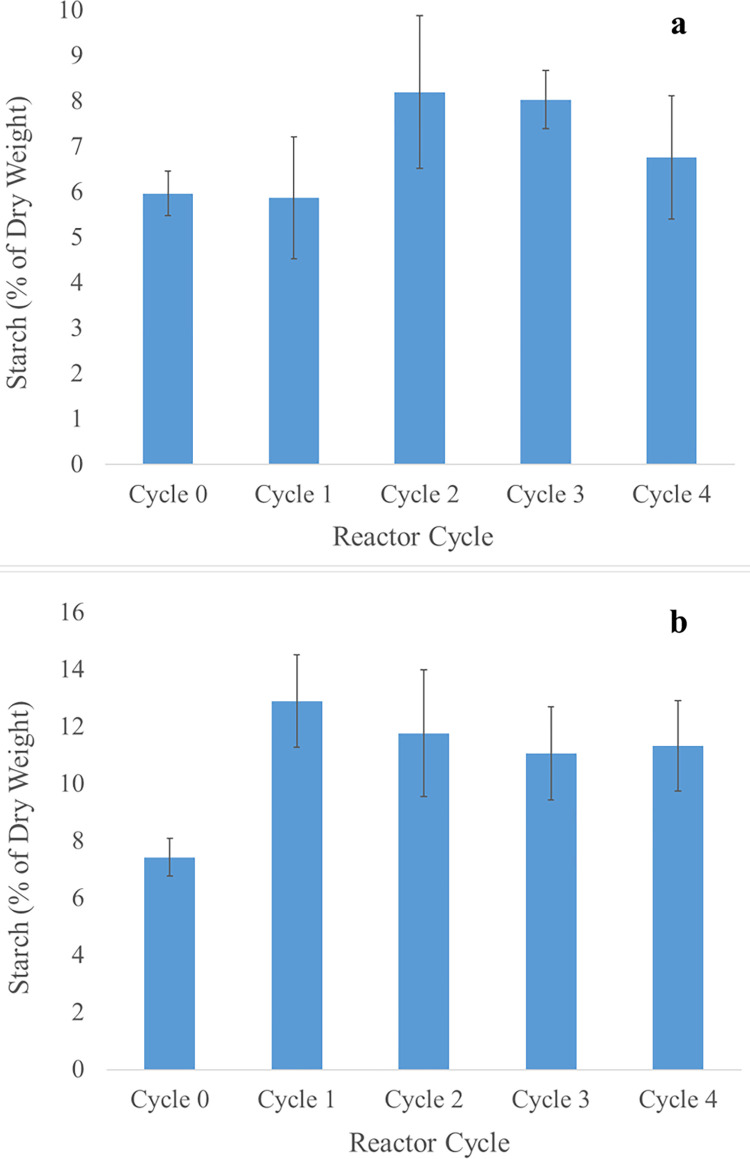
Carbohydrate storage in algal biomass. Starch content of a) pH 9 and b) pH 7.5 reactors over the course of feast-famine cycles. Error bars represent one standard deviation above and below of three biological triplicates.

The starch content of reactor biomass did not change significantly with feast-famine cycling, with the exception of an increase in average starch content in pH 7.5 reactors from 7.4% to 12.9% after the first feast-famine cycle (Fig 2). Overall, the average starch content of the pH 7.5 reactors was higher (ranging from 7.4% to 11.7%) than that of the pH 9 reactors (ranging from 6.0% to 8.0%). Our results suggest that feast-famine cycling in simulated secondary wastewater effluent did not produce a selection pressure on microalgae for the accumulation of any form of reduced carbon during periods of oligotrophy (as either starch or lipids).

### Biomass concentration and total 16S abundance

**Fig 3:**
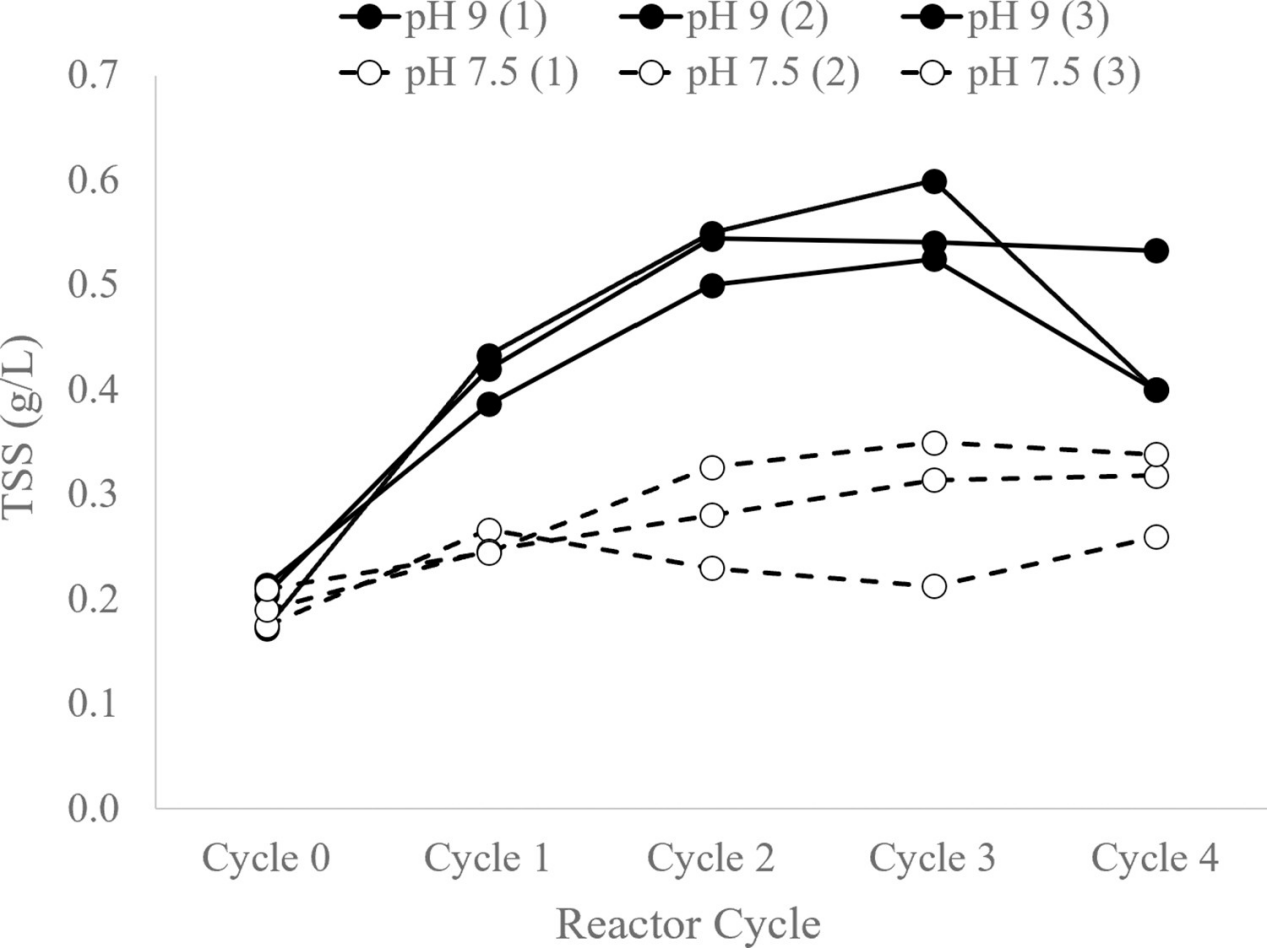
Reactor biomass concentrations. TSS concentration (g/L) in pH 7.5 and pH 9 reactors over the course of nutrient cycles for biological triplicate reactors.

The total suspended solids (TSS) in reactors increased from an average of 0.2 ± 0.02 g/L to a final average TSS of 0.4 ± 0.08 g/L at pH 9 and from 0.2 ± 0.02 g/L to 0.3 ± 0.04 g/L at pH 7.5 (Fig 3), indicating net biomass growth during the experiment. TSS increased or remained stable across reactors for each cycle, except for decreases in two of the pH 9 reactors between Cycles 3 and 4 and one of the pH 7.5 reactors between Cycles 1 and 3. Additionally, the TSS in the pH 9 reactors was consistently higher than the TSS in the pH 7.5 reactors across all reactor cycles.

**Fig 4:**
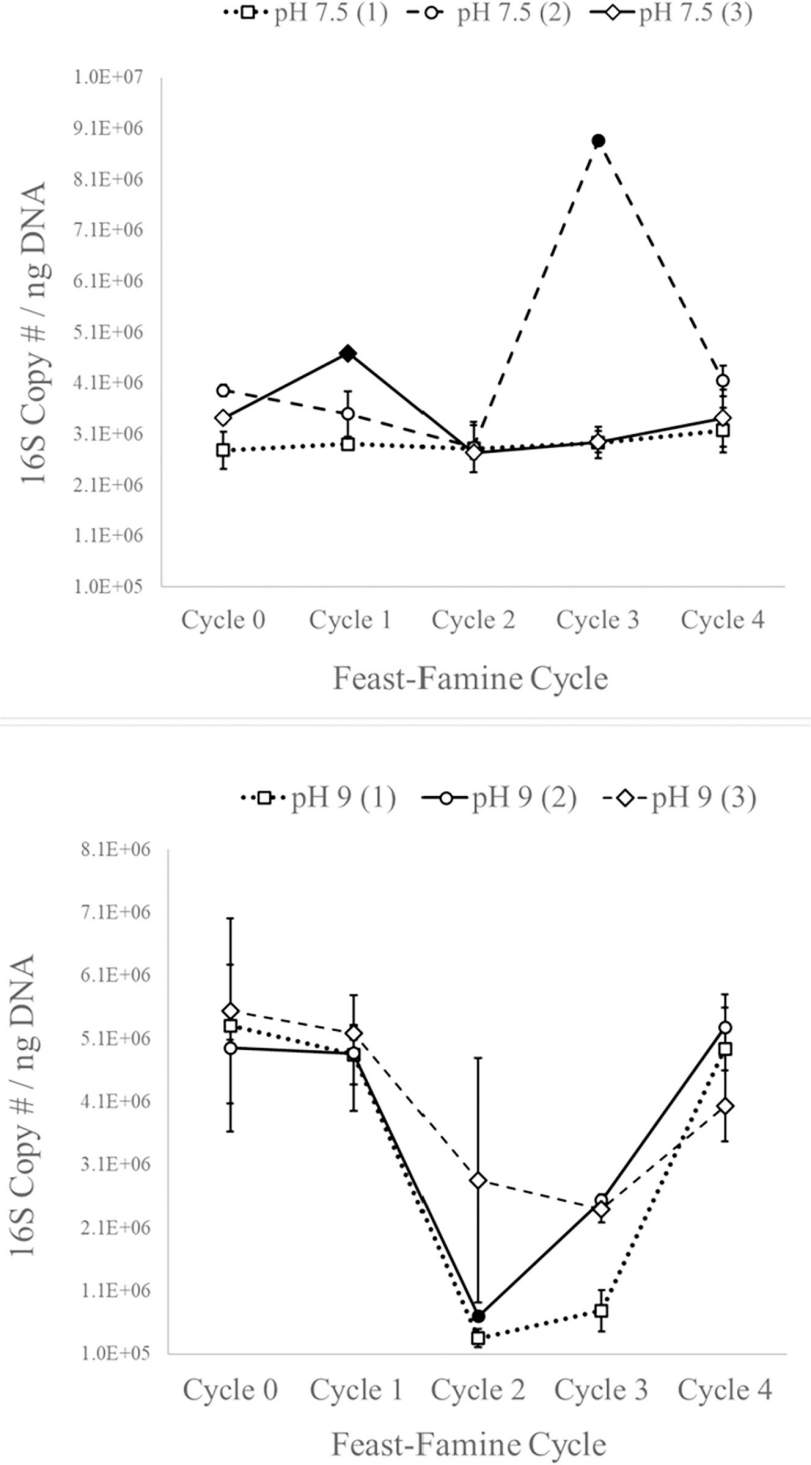
Total 16S rRNA gene copy number normalized to ng DNA. Indication of bacterial DNA to total DNA ratio for biological triplicate reactors at pH 9 and pH 7.5. Error bars represent range of duplicate readings (solid filled data point indicates a sample for which only one reading was available).

Total bacterial abundance was measured in the bioreactors over feast-famine cycles by qPCR of total 16S rRNA gene copy number normalized to extracted DNA concentration, in order to estimate the proportion of bacteria versus eukaryotic organisms in the total biomass. The 16S rRNA gene abundance as a share of total biomass DNA decreased as a result of feast-famine cycling for pH 9 reactors between cycles 0 and 3, and it subsequently increased again between cycles 3 and 4 (ANOVA P < 0.001) (Fig 4). For pH 7.5 reactors, there was no statistically significant change in 16S abundance (ANOVA P = 0.617).

Since bacteria generally accumulate less lipids than algae, an increase in the overall proportion of bacteria relative to total biomass in the reactors could lead to a decrease in biomass lipid content. The bacteria measured by 16S rRNA gene copy number in the reactors includes cyanobacteria, which can accumulate lipids, but at a lesser extent than green algae [31, 32].

The overall decrease in lipid content over time in pH 9 reactors (Fig 1) does not appear to be the result of bacteria outcompeting green algae because there is no corresponding increase in total 16S rRNA gene copy number as a result of feast-famine cycling. In addition, there is no direct relationship between bacterial abundance and lipid content in the pH 9 reactors, since 16S rRNA gene copy number decreased between cycles 0 and 1 while lipid content increased in pH 9 reactors over this period.

### Microbial community composition

**Fig 5:**
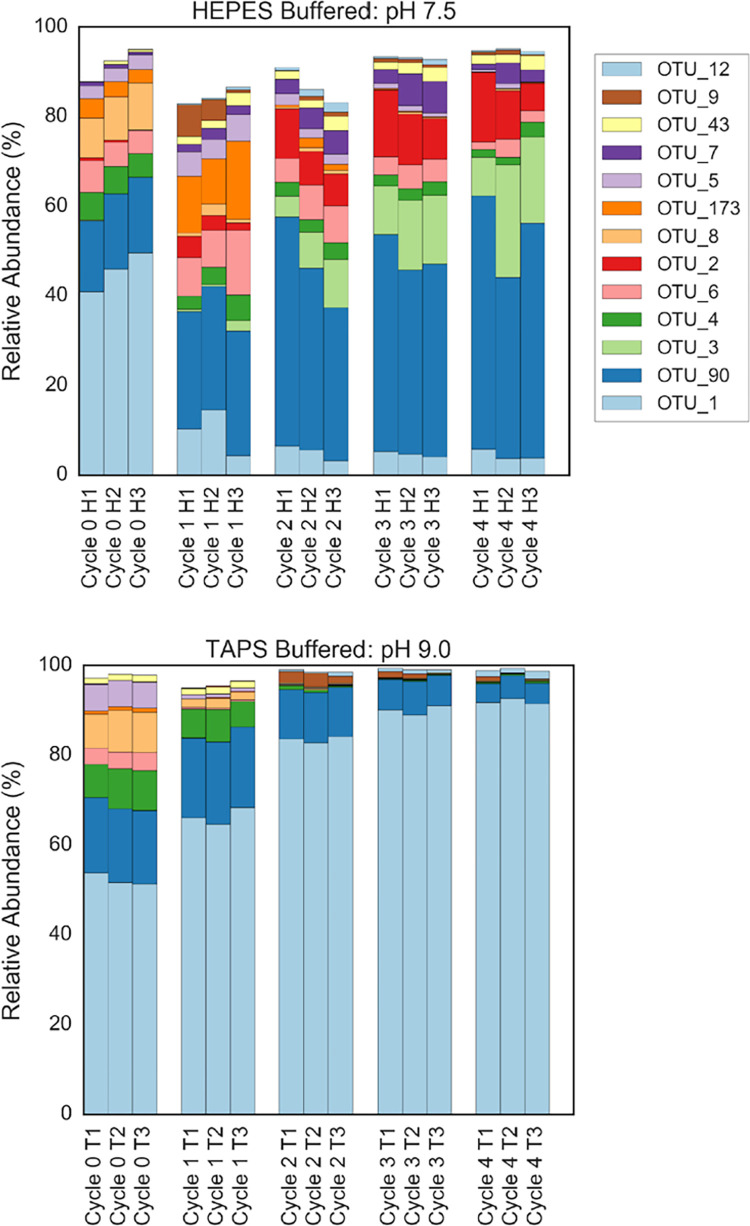
Microbial community composition shifts in reactors. Eukaryotic microbial community composition inferred from 18S rRNA gene sequencing in pH 7.5 reactors and pH 9 reactors, sampled at the first four resuspension (OTUs with average abundance <0.5% removed). H1, H2, and H3 and T1, T2, and T3 represent biological triplicate reactors buffered with HEPES (pH 7.5) and TAPS (pH 9), respectively.

Eukaryotic microbial community compositions as described by 18S rRNA gene OTU profiles in the pH 7.5 and pH 9 reactors were initially similar (Weighted Unifrac Distance between communities at Cycle 0, ANOSIM R = 0.59, P = 0.96), but by the end of the first feast-famine cycling, the composition in the two sets of reactors began to diverge, while replicates of reactors maintained close similarity (Fig 5). Two highly represented OTUs aligning to the *Scenedesmus* or *Desmodesmus* genus (OTUs: 1, 90) accounted for a large percentage of total reads in the samples across cycles for pH 7.5 reactors (32–67%) as well as pH 9 reactors (68–98%). This large amount of *Scenedesmus*/*Desmodesmus* aligned reads is consistent with microbiome analyses in other photobioreactors [33].

**Fig 6:**
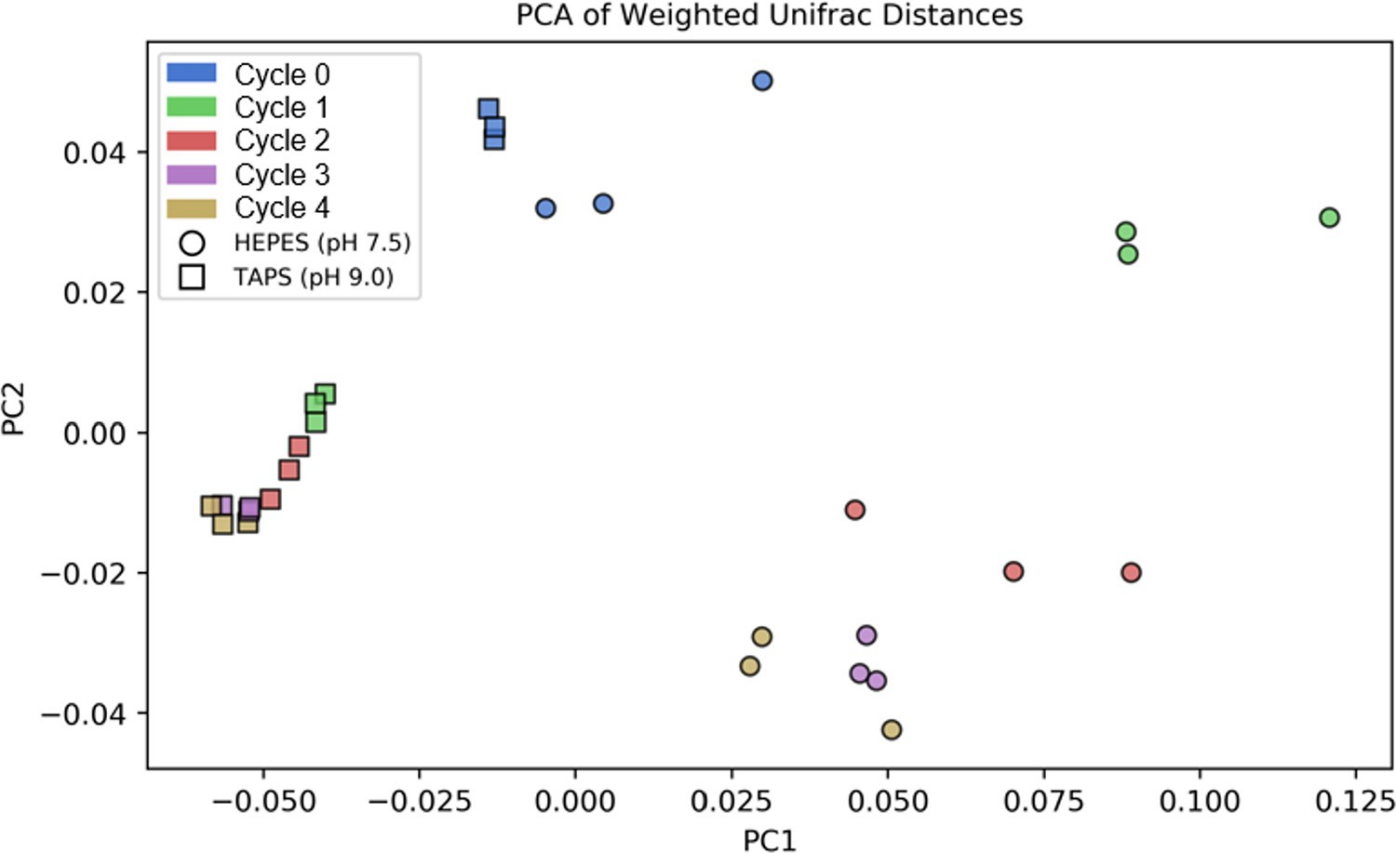
Principal component analyses of microbial community data. Weighted Unifrac Distances for 18s rRNA gene amplicon sequencing data showing the pH 9 reactors (squares) and pH 7.5 reactors (circles). Cycle number is indicated by color.

**Fig 7:**
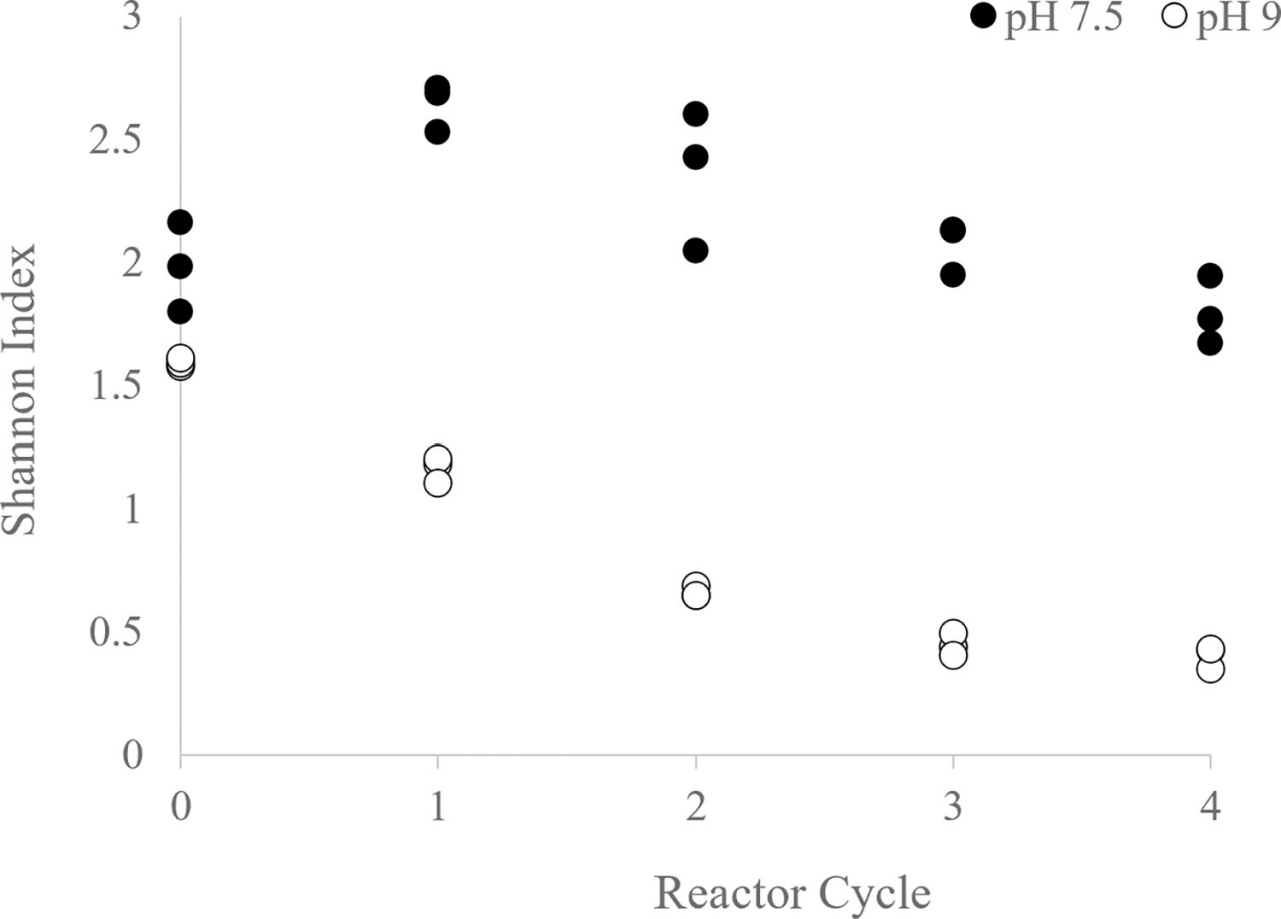
Shannon index (H) of pH 9 and pH 7.5 reactors. Change in H over several reactor cycles.

Statistically significant changes in lipid content were detected for pH 9 reactors but not pH 7.5 (Fig 1), and the microbial communities diverged different between these sets of reactors (Weighted Unifrac Distance between week 1–4 communities, ANOSIM R = 0.91, P < 0.001). Communities in both pH treatments underwent succession and adaptation to reactor conditions when viewed in terms of overall beta diversity (Fig 6). The pH 9 reactors rapidly converged to a community dominated by a single *Desmodesmus-*affiliated OTU (OTU_1) within the first feast-famine cycle. The abundance of OTU_1 in pH 9 reactors increased monotonically throughout the study period (Fig 7). The pH 7.5 reactors had higher week-to-week variability but displayed consistent community dynamics across replicate reactors. These reactors showed selection for the *Scenedesmus*/*Desmodesmus* aligned OTU_90. Our results suggest that physiological differences between OTU_1 and other algal OTUs present in the community could account for changes in lipid content in this reactor over time.

Competitive exclusion of other organisms by OTU_1 or strong environmental selection imposed by the elevated pH could explain decreased bacterial abundances as well as a decrease in Shannon Index diversity in pH 9 reactors, which was not seen in pH 7.5 reactors (Fig 7). The decrease in diversity in the pH 9 reactor could have contributed to physiological shifts and net decreases in lipid content over feast-famine cycles in these reactors, as lipid content of microalgal communities have been shown to increase with increasing species richness [34].

In pH 9 reactors, OTU_1 was a dominant community member initially at Cycle 0, representing half of all sequencing reads. Between Cycle 0 and Cycle 1, OTU_1 increased in abundance to account for an additional 13.5% of reads and thus appeared to be primarily responsible for displacing other OTUs that had decreased in abundance over this cycle. This shift in community composition corresponded to an increase in average lipid content from 12.2% to 16.2%. Between Cycle 1 and Cycle 4, OTU_1 increased in abundance further, however this time in conjunction with a decreasing overall lipid biomass content in reactors, down to 5.2% lipids by dry weight at Cycle 4. Dominant OTU_1 displacing other OTUs, then, corresponded to both an increase and decrease in overall lipid content of the cultures. A change in the physiological state of algae as a result of feast-famine cycling to produce a lower lipid content appears necessary to reconcile the shifts in lipid composition with shifts in microbial community composition.

Microbial community profiling results show that feast-famine cycling selected for *Desmodesmus/Scenedesmus* at both pH 7.5 and pH 9. *Scenedesmus* in another study was shown to perform best for the dual purposes of wastewater nutrient removal and lipid accumulation when compared to other laboratory strains [35]. Although this *Scenedesmus/Desmodesmus* consortia in our reactors did not have a higher lipid content than the initial inoculum, the ability to select strongly for this OTU has implications for biotechnology applications of algae. Modifications to the specific parameters of SBR operation may alter selection pressures towards an algal community with higher lipid productivity (or other desired functionality). Alternatively, combining a feast-famine selection pressure with bioaugmentation of a strain of *Desmodesmus* or *Scenedesmus* with a favorable functionality such as high lipid content may allow for the bioaugmented strain to be maintained against invasion by other strains over time.

One potential application of the results of feast-famine cycling from our study could be genetically engineering a strain of *Desmodesmus* for a desirable degradation pathway of an organic wastewater contaminant. A study of microalgal bioaugmentation revealed a beneficial impact of the addition of *H*. *pluvialis* on membrane bioreactor performance and antibiotic degradation [36]. Application of feast-famine cycling at high pH could then preferentially maintain the presence of this desirable engineered trait in reactors by depressing competition and species diversity.

Overall, our results suggest that cultivation of algae under feast-famine conditions fed with secondary wastewater could result in the selection for biomass with low lipid content. However, other explanations for the effects of feast-famine cycling on the simulated secondary wastewater effluent are possible. Synthetic secondary wastewater effluent was a chemically defined media, and thus feast-famine cycling may have simply selected for algal species with a proclivity for rapid uptake of a limiting nutrient species (such as nitrate or phosphate). However, real secondary wastewater effluent would still likely be dominated by similar nutrients, especially at plants with nitrification which would convert TKN into predominately nitrate. It is also possible that a different cycling time could lead to different selection pressures on the algal community. Finally, the ultimate effects of feast-famine cycling could be dependent on the community composition of algae at the beginning of reactor operation (since reactors in this study were operated aseptically, thus limiting microbial immigration to fill ecological niches). For this reason, limited initially diversity at the time of reactor operation is also an alternative explanation for our results regarding the effects of feast-famine cycling on reactors.

Higher lipid contents in cultivated algae will result in more favorable economics for algal biofuel production and several options are under consideration to optimize algal lipid content. Generally, one approach could be based on using engineered mixed algal community assembly through the selection of strains with complementary and desirable traits for inoculation into algal bioreactors [37]. Alternatively, another approach is to create a selective environment during biomass cultivation which favors desired traits, thus creating sustained ecological pressures which select for favorable attributes out of a natural, diverse pool of microorganisms [38]. Our results provide insight into the latter of these two approaches to optimizing algal biomass lipid content, indicating that where feast-famine selection has worked for creating desirable selection pressures in other bioengineering applications (e.g., PHB production in bacteria), it is not necessarily the case for algal lipid production. More research and study will be required to examine if changes to operating parameters can make a feast-famine selection pressure viable, or if other selection approaches should be examined instead.

While our results have implications for imposed stresses on bioreactor-cultivated algae, they also may be relevant to natural systems which experience varying nutrient concentrations. The combinations of variables used in these experiments produced decreasing species diversity in feast-famine reactors, and thus could produce decreasing species diversity in natural environments. This would have implications for the ecosystem stability, which generally has a positive relationship with diversity [39].

## Conclusions

In conclusion, feast-famine reactor cycling had a neutral or decreasing effect on lipid content of algal biomass in bioreactors, and the imposed reactor cycling did affect microbial community structure. Effects of feast-famine cycling on eukaryotic microbial communities were pH dependent, and at pH 9 resulted in the selection of a single OTU that dominated reactor biomass by 18S rRNA gene sequence count. This selection corresponded to a decrease in species diversity in the bioreactor along with a decrease in the lipid content of biomass in this bioreactor.

## Supporting information

S1 Table Quality check of modified starch extraction method. Repeatability in replicates of flour standards and algal samples as indicated by measured % starch by dry weight (% dw) using scaled-down reaction volumes.
(DOCX)

S1 Fig Linearity check of modified starch extraction method. Test of linearity of lower-volume Megazyme starch kit analysis using a glucose standard curve.
(DOCX)
