# Supplementary figures and images for: Effects of feast-famine nutrient regimes on wastewater algal biofuel communities
Source: PLoS One. 2023 Jan 4;18(1):e0279943. doi: 10.1371/journal.pone.0279943 (PMC9812324; doi:10.1371/journal.pone.0279943)

**Supporting Information**

**S1 Table**


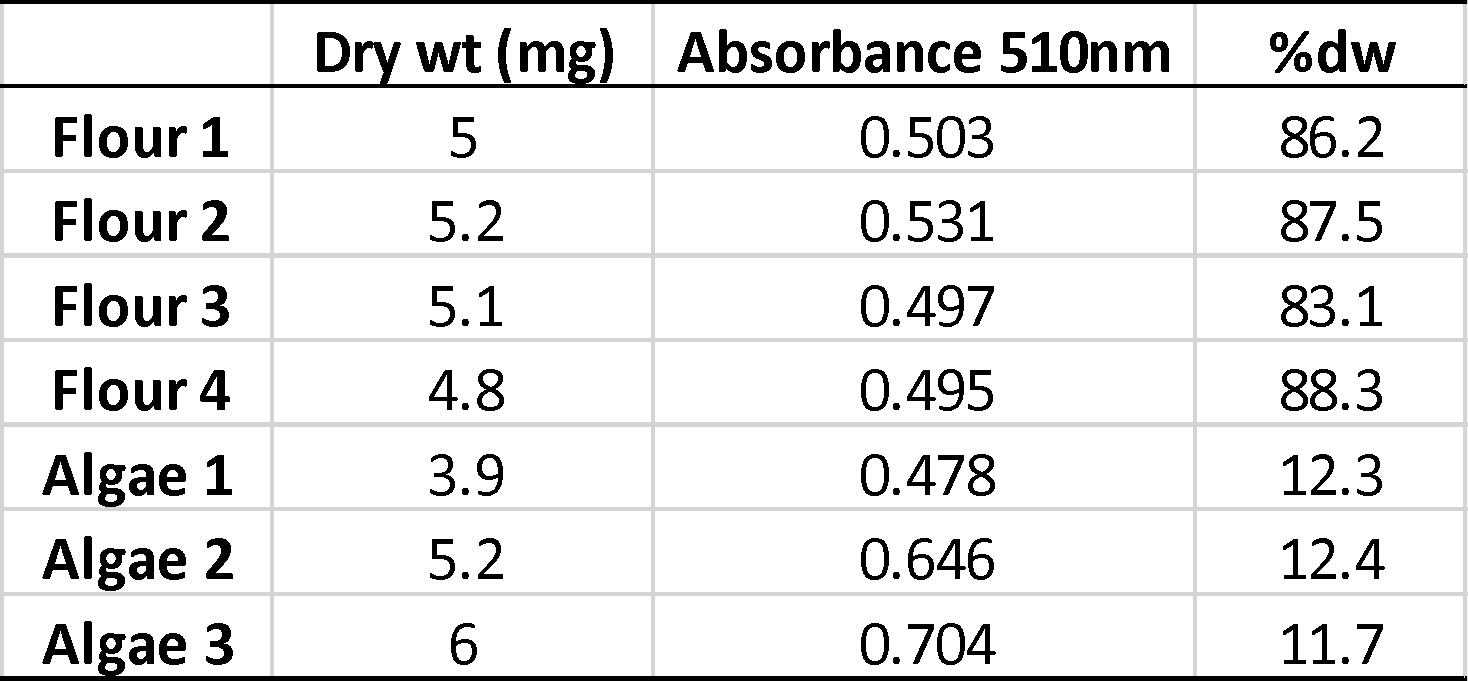

Supplement: S1 Table — Repeatability in replicates of flour standards and algal samples as indicated by measured % starch by dry weight (% dw) using scaled-down reaction volumes. (DOCX) [file pone.0279943.s001.docx]

**Supporting Information**


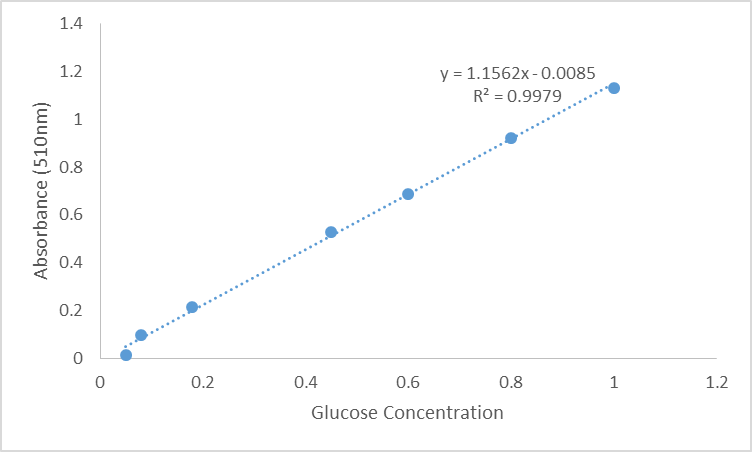


**S1 Fig**

Supplement: S1 Fig — Test of linearity of lower-volume Megazyme starch kit analysis using a glucose standard curve. (DOCX) [file pone.0279943.s002.docx]
